# Global Status of DDT and Its Alternatives for Use in Vector Control to Prevent Disease

**DOI:** 10.1289/ehp.0900785

**Published:** 2009-05-29

**Authors:** Henk van den Berg

**Affiliations:** Laboratory of Entomology, Wageningen University, Wageningen, the Netherlands

**Keywords:** DDT, indoor residual spraying, integrated vector management, malaria, persistent organic pollutants, vector control

## Abstract

**Objective:**

I review the status of dichlorodiphenyltrichloroethane (DDT), used for disease vector control, along with current evidence on its benefits and risks in relation to the available alternatives.

**Data sources and extraction:**

Contemporary data on DDT use were largely obtained from questionnaires and reports. I also conducted a Scopus search to retrieve published articles.

**Data synthesis:**

DDT has been recommended as part of the arsenal of insecticides available for indoor residual spraying until suitable alternatives are available. Approximately 14 countries use DDT for disease control, and several countries are preparing to reintroduce DDT. The effectiveness of DDT depends on local settings and merits close consideration in relation to the alternatives. Concerns about the continued use of DDT are fueled by recent reports of high levels of human exposure associated with indoor spraying amid accumulating evidence on chronic health effects. There are signs that more malaria vectors are becoming resistant to the toxic action of DDT, and that resistance is spreading to new countries. A comprehensive cost assessment of DDT versus its alternatives that takes side effects into account is missing. Effective chemical methods are available as immediate alternatives to DDT, but the choice of insecticide class is limited, and in certain areas the development of resistance is undermining the efficacy of insecticidal tools. New insecticides are not expected in the short term. Nonchemical methods are potentially important, but their effectiveness at program level needs urgent study.

**Conclusions:**

To reduce reliance on DDT, support is needed for integrated and multipartner strategies of vector control and for the continued development of new technologies. Integrated vector management provides a framework for developing and implementing effective technologies and strategies as sustainable alternatives to reliance on DDT.

The Stockholm Convention seeks the elimination of 12 chemicals or classes of chemicals, one of which is dichlorodiphenyltrichloroethane (DDT) [United Nations Environment Programme (UNEP) 2002]. DDT is used in indoor spraying for control of vectors of malaria and visceral leishmaniasis. In negotiations that led to the treaty, there was concern that a sudden ban on DDT use could adversely affect the malaria burden. Thus, DDT was permitted to be produced and used for the purpose of controlling disease vectors in accordance with recommendations and guidelines of the World Health Organization (WHO) and when locally safe, effective, and affordable alternatives are not available (WHO 2007a). Ironically, DDT use in Africa has increased since the Stockholm Convention came into effect (Manga L, personal communication).

Malaria is a complex parasitic disease confined mostly to tropical areas and transmitted by mosquitoes of the genus *Anopheles*. There are an estimated 250 million clinical cases of malaria, causing nearly a million deaths, mostly of children < 5 years of age and mostly in sub-Saharan Africa (WHO 2008b). Malaria-endemic countries are faced with a high cost of prevention and treatment of the disease.

Vector control is an essential component of malaria control programs. The WHO has reaffirmed the importance of vector control through indoor residual spraying (IRS) as one of the primary interventions for reducing or interrupting malaria transmission in countries in both stable and unstable transmission zones. Twelve insecticides have been recommended for IRS, including DDT. The course of action promoted by the WHO has been to retain DDT as part of the arsenal of insecticides available for IRS globally, to be able to manage insecticide resistance until suitable alternatives are available (WHO 2007a). The use of DDT for IRS is recommended only where the intervention is appropriate and effective in the local epidemiologic situation. Nonetheless, DDT has not been subjected to the WHO’s Pesticide Evaluation Scheme for many years.

In this review, I present the current situation regarding the use of DDT for vector control, covering aspects of production, use, legislation, cost-effectiveness, health effects, environmental effects, insecticide resistance, monitoring, and evaluation. I provide an outline of alternative methods, strategies, and new developments; discuss cost-effectiveness, current implementation, barriers, and gaps in implementing the alternatives; and present possible solutions to reduce reliance on DDT.

This review is based largely on a document commissioned by the Stockholm Convention Secretariat, which served as background paper for a global stakeholders’ meeting to review the establishment of a global partnership to develop alternatives to DDT, held 3–5 November 2008 in Geneva, Switzerland.

## Methods

Contemporary information on the production and use of DDT was obtained from *a*) formal questionnaires by the Stockholm Convention Secretariat, completed by national authorities; *b*) documents published by the Stockholm Convention; *c*) direct communications with national authorities; and *d*) information available from project proposals submitted to the Global Environment Facility (2009). Information has been supplemented with data presented by country delegates at workshops in the context of the Stockholm Convention.

I obtained information on side effects, insecticide resistance, cost-effectiveness, and alternatives from literature searches. I used the search engine Scopus (2008) to retrieve studies related to DDT and malaria, with vector control as additional search term. Because of the breadth of the subject matter, only the most relevant studies were selected, and reviews were prioritized. Old literature was accessed electronically, or hard copies were obtained from libraries. Additional information on insecticide resistance was obtained from web-based reports from the African Network on Vector Resistance (ANVR) (Vector Biology and Control 2008). Information on human exposure and health effects was based on reviews published over the past 5 years and supplemented with recent studies on exposure due to indoor spraying.

## Status of DDT

### Production, use, and management

DDT is currently being produced in three countries: India, China, and the Democratic People’s Republic of Korea (DPRK; North Korea) (Table 1). By far the largest amounts are produced in India for the purpose of disease vector control. In China, the average annual production during the period 2000–2004 was 4,500 metric tons of DDT, but 80–90% was used in the production of Dicofol, an acaricide, and around 4% was used as additive in antifouling paints. The remainder was meant for malaria control and was exported. Recent information from the DPRK [United Nations Institute for Training and Research (UNITAR), unpublished data] indicates that 160 metric tons of DDT is produced per year, for use mainly in agriculture (which is not acceptable under the Stockholm Convention) and a small portion for use in public health. India and China both export DDT to countries in Africa, either as technical product or as a formulation, for the purpose of vector control. DDT is being formulated in Ethiopia and South Africa with ingredients imported from China. South Africa exports some of its formulated product to other countries in Africa.

An estimated 5,000 metric tons of DDT (active ingredient) was used for disease vector control in 2005 (Table 1). The primary use is for malaria control, but approximately 1,000 metric tons/year (20% of global consumption) is used for control of visceral leishmaniasis restricted to India. India is by far the largest consumer of DDT, but in 2007 use was down one-fourth from the 2005 level. Mozambique, Zambia, and Zimbabwe have recently reintroduced the use of DDT. With the possible exception of the Dominican Republic, there is no reported use of DDT for disease vector control from the Americas. Use in Ecuador, Mexico, and Venezuela was phased out in 2000. China has reported that no DDT has been used for disease vector control since 2003, and future use is reserved only for malaria outbreaks.

IRS programs are currently expanding in Africa, the main driver being the U.S. President’s Malaria Initiative (PMI 2009). Pilot programs on IRS have been initiated in some African countries, and several other countries are considering reintroducing the intervention. In some of these countries, a decision has not been made on whether to use DDT in their IRS program. Hence, the use of DDT may be increasing—especially in African countries—because new countries are initiating IRS programs, including the use of DDT, and countries that are using DDT are expanding their IRS programs to stable transmission areas.

There is a paucity of data on DDT supplies. The available information indicates that large amounts of DDT are stored in many countries, but most of the stock is outdated or of unknown quality. Moreover, the transfer of DDT stock between countries is not always documented or reported, and this poses a problem in tracking quantities of the chemical and establishing the quality of DDT being used. A major multistakeholder effort is needed for the cleanup of outdated DDT stock, for example, through the Africa Stockpiles Programme (Curtis and Olsen 2004).

Many countries that use DDT have inadequate legislation or lack capacity to implement or enforce regulations on pesticide management. Unpublished information suggests that DDT is being traded on local markets for use in agriculture and termite control (UNEP 2008). Funding agencies aiding in the purchase of DDT should be obligated to provide financial assistance to ensure that regulations and monitoring capacity are in place to support proper management of DDT from the cradle to the grave, for example, by involving the environmental sector.

### Cost-effectiveness of DDT

No published data exist on cost-effectiveness in terms of cost per disability-adjusted life-year averted by IRS using DDT. Statements of high cost-effectiveness of DDT have been based on the positive experience from the malaria eradication era (Mabaso et al. 2004) supplemented with more recent results on reductions in malaria morbidity and incidence associated with the use of DDT (Curtis 2002; Gunasekaran et al. 2005; Sharp et al. 2007).

Both the effectiveness and costs of DDT are dependent on local settings and merit careful consideration in relation to alternative products or methods. DDT has been known as the only insecticide that can be used as single application in areas where the transmission season is > 6 months. However, information is lacking on the potential variability in residual action of insecticides, including DDT (e.g., due to sprayable surface, climatic conditions, social factors).

Direct costs of IRS are the procurement and transport of insecticide, training of staff, operations, awareness-raising of communities, safety measures, monitoring of efficacy and insecticide resistance, monitoring of adverse effects on health and the environment, and storage and disposal. In 1990, the insecticide costs per house per 6 months of control were substantially lower for DDT (US$1.60) than for other insecticides (> US$3.40), but in 1998 the cost range for DDT (US$1.50–3.00) overlapped with that of alternative insecticides (> US$2.20), pyrethroids in particular (Walker 2000). This comparison will further change with the availability of new formulations of pyrethroids that have increased residual activity. Moreover, incorporating the cost of safety measures in the application of DDT will significantly change its comparative cost advantage.

Apart from the direct costs, it is essential that the unintended costs of DDT (or alternative insecticides) to human health and the environment are included in the cost assessment. In addition, contamination of food crops with DDT could negatively affect food export (Anonymous 2007). A comprehensive cost assessment of DDT versus its alternatives should include the potential costs of atmospheric transport and chronic health effects.

Proposed and ongoing projects by the WHO, United Nations Environment Programme, and United Nations Development Programme are expected to establish a more solid evidence base for the effectiveness of DDT in relation to its alternatives (WHO 2007b). The results will be crucial in future decision making on vector management strategies for prevention of malaria.

### Health effects of DDT

High levels of human exposure to DDT among those living in sprayed houses, most of whom are living under conditions of poverty and often with high levels of immune impairment, have been found in recent studies in South Africa and Mexico (Aneck-Hahn et al. 2007; Bouwman et al. 1991; De Jager et al. 2006; Yanez et al. 2002), but contemporary peer-reviewed data from India, the largest consumer of DDT, are lacking. The simultaneous presence of, and possible interaction between, DDT, dichlorodiphenyldichloroethylene (DDE), and pyrethroids in human tissue is another area of concern (Bouwman et al. 2006; Longnecker 2005). In North America, rather high levels of exposure have been recorded in biological samples collected near the time of peak use during the 1960s (Eskenazi et al. 2009). Exposure of the fetus and young child occurs through the placenta and through lactation (Bouwman et al. 2006); exposure of children and adults occurs through direct contact with DDT in the environment, through indoor dust (Herrera-Portugal et al. 2005), and through the food chain. DDT accumulates in fatty tissue and is slowly released. A monitoring system is needed for the assessment of trends in exposure to DDT, allowing for the attribution of effects to IRS locally; in this regard, human milk is considered an important media to be monitored (Malisch and van Leeuwen 2003).

Studies on health effects of DDT have focused mostly on subjects in North America and Europe, who have generally been exposed to levels lower than those reported from areas with IRS. No global assessment has been made on the evidence of health risks of DDT in relation to IRS because data are scarce. As an indication, however, initial work suggests that nonoccupational exposure through IRS is associated with impaired semen quality in men (Aneck-Hahn et al. 2007; De Jager et al. 2006).

Health effects of DDT and DDE most commonly suggested by studies in North America and Europe are early pregnancy loss, fertility loss, leukemia, pancreatic cancer, neurodevelopmental deficits, diabetes, and breast cancer (Beard 2006; Chen and Rogan 2003; Cox et al. 2007; Eriksson and Talts 2000; Garabrant et al. 1992; Ribas-Fito et al. 2006; Snedeker 2001; Venners et al. 2005). In many cases the results have not been consistent between studies, but nevertheless these accumulating reports bear much concern, particularly in relation to chronic effects. Breast cancer has been most rigorously studied; even though the majority of results showed no causative association with DDT exposure (Brody et al. 2007), the latest evidence indicates an increased risk in women who were exposed at a young age (Cohn et al. 2007). In addition, experimental studies on animals have demonstrated neurotoxic, carcinogenic, immunotoxic, and reproductive effects attributable to DDT and DDE (Turusov et al. 2002).

The adverse health effects of DDT versus the health gains in terms of malaria prevention require more attention. For example, a gain in infant survival resulting from malaria control could be partly offset by an increase in preterm birth and decreased lactation, both of which are high risk factors for infant mortality in developing countries. The WHO is conducting a reevaluation of health risks of DDT, but progress has been slow.

### Environmental effects of DDT

As a persistent molecule, DDT has low to very low rates of metabolism and disposition, depending on ambient temperatures. It is degraded slowly into its main metabolic products, DDE and dichlorodiphenyldichloroethane (DDD), which have similar physicochemical properties but differ in biological activity. DDT is emitted through volatilization and runoff. It is more volatile in warmer than in colder parts of the world, which through long-range atmospheric transport results in a net deposition and thus gradual accumulation at high latitudes and altitudes (Harrad 2001).

Loss through runoff is low because DDT has a strong affinity for organic matter in soils and aquatic sediment but is virtually insoluble in water. Half-lives of DDT have been reported in the range of 3–7 months in tropical soils (Varca and Magallona 1994; Wandiga 2001) and up to 15 years in temperate soils (Ritter et al. 1995). The half-life of each of its metabolic products is similar or longer. DDT readily binds with fatty tissue in any living organism, and because of its stability, bioconcentrates and biomagnifies with increasing trophic level in food chains (Kelly et al. 2004). The half-life of DDT in humans is > 4 years; the half-life for DDE is probably longer (Longnecker 2005). Studies have shown that DDT is highly toxic to insects, shrimp, and fish (Fisk et al. 2005; Galindo et al. 1996; Metcalf 1973) and adversely affects the reproduction of wild birds through thinning of egg shells (Ratcliffe 1967).

DDT and its metabolic products present in the global environment have originated mostly from its previous large-scale use in agriculture and domestic hygiene. Because DDT is currently allowed only for indoor spraying for disease vector control, its use is much smaller than in the past. Nevertheless, DDT sprayed indoors may end up in the environment (e.g., when mud blocks of abandoned houses are dissolved in the rain). Data from Brazil, India, Mexico, and South Africa suggested that higher levels of DDT are found in water or soil samples in areas with DDT residual spraying than in areas without spraying (Bouwman et al. 1990; Dua et al. 1996; Sereda and Meinhardt 2005; Vieira et al. 2001; Yanez et al. 2002), but these results need further verification.

### Insecticide resistance

As the number and size of programs that use DDT for indoor spraying increase, insecticide resistance is a matter of growing concern. Since the introduction of DDT for mosquito control in 1946, DDT resistance at various levels has been reported from > 50 species of anopheline mosquitoes, including many vectors of malaria (Hemingway and Ranson 2000). Unless due attention is paid to the role of insecticide resistance in the breakdown of the malaria eradication campaign of the 1960s, resistance may once again undermine malaria control (Busvine 1978).

In the past, the use of DDT in agriculture was considered a major cause of DDT resistance in malaria vectors, as many vectors breed in agricultural environments (Mouchet 1988). At present, DDT resistance is thought to be triggered further by the use of synthetic pyrethroids (Diabate et al. 2002). This is due to a mechanism of cross-resistance between pyrethroids and DDT, the so-called sodium channel mutation affecting neuronal signal transmission, which is governed by the *kdr* (knock-down resistance) gene (Martinez-Torres et al. 1998). Vectors with the *kdr* gene are resistant to both groups of insecticides, and this has serious consequences for malaria vector control, because pyrethroids and DDT are the two main groups of chemicals used. The *kdr* gene is being reported from an increasing number of countries; thus, even in countries without a history of DDT use, resistance to DDT is emerging in populations of malaria vectors (WHO 2006).

Contemporary data from sentinel sites in Africa indicate that the occurrence of resistance to DDT is widespread, especially in West and Central Africa (ANVR 2005; Coleman et al. 2007). The main African vector, *Anopheles gambiae s.s.,* showed resistance to DDT in the majority of tests. Further, there is recent evidence of resistance in *A. gambiae s.l.* in Ethiopia (ANVR 2005), and there are signs of DDT resistance in *Anopheles arabiensis*, another key vector, from Uganda, Cameroon, Sudan, Zimbabwe, and South Africa. In Asia, the resistance to DDT is particularly widespread in India. Multiple resistance to DDT and other insecticides in the major vector *Anopheles culicifacies* is present in many parts of the country (Dash et al. 2009) and has reportedly caused a major loss in effectiveness of intervention (Sharma 2003). Resistance has also been reported in *Anopheles sinensis* from China (Cui et al. 2006) and in *Anopheles epiroticus* (formerly named *Anopheles sundaicus*) in Vietnam (Dusfour et al. 2004).

Resistance does not necessarily result in failure to control disease. Standard testing of DDT resistance focuses on the insecticide’s toxic action. However, the repellent and irritant properties of DDT also have the potential to reduce transmission of disease and relieve the selective pressure for toxic resistance (Grieco et al. 2007; Roberts and Andre 1994). This is an area requiring more research.

An important lesson learned from the experience with oncocerciasis (river blindness), another vector-borne disease, is that the development and spread of insecticide resistance is much slower when vector populations are under effective control (Guillet P, personal communication), suggesting that suppressing vector proliferation helps prevent or delay the development of resistance.

Effective monitoring and decision support systems can enable insecticide resistance to be detected at an early stage, which should lead to the implementation of changes in insecticide policy (Sharp et al. 2007). However, the choice of unrelated insecticides remains limited (Nauen 2007). Even an intelligent insecticide resistance management strategy using rotations, mosaics, or mixtures may not prevent resistance development (Hemingway et al. 1997; Penilla et al. 2006). In a recent report from India, the Joint Monitoring Mission (JMM 2007) pointed out that the insecticide choice for IRS is rarely based on contemporary insecticide susceptibility testing.

## Alternatives to DDT

A number of vector control methods are available as alternatives to DDT. Two of these, the use of alternative insecticides in IRS and the use of insecticide-treated bed nets (ITNs), are mainstreamed because of their proven impact on the malaria burden. Other available alternatives are receiving limited attention in contemporary malaria control efforts, but have an important role to play. Table 2 summarizes alternative methods. Alternatives to DDT should pose less risk to human health and the environment and be supported with monitoring data.

### Chemical methods

IRS with insecticides is an effective method of malaria control. Its strength lies in its effect on shortening the life span of adult mosquitoes near their human targets, which has a critical impact on malaria transmission (MacDonald 1957). However, there is limited information on effectiveness and operational feasibility of IRS in African countries with highly endemic malaria, some of which recently reintroduced IRS or plan to do so. Twelve insecticides belonging to four chemical classes are recommended for IRS in vector control, which collectively address only three modes of toxic action (Nauen 2007). Pyrethroids are the most cost-effective alternatives to DDT in malaria control except where pyrethroid resistance occurs (Walker 2000).

There are two new developments with regard to IRS. First, some existing insecticides not currently available for public health; chlorfenapyr and indoxacarb, for example, showed potential in areas with pyrethroid resistance (N’Guessan et al. 2007a, 2007c). Second, new formulations of existing insecticides with prolonged residual activity are being developed as alternatives to DDT (Hemingway et al. 2006). Two slow-release formulations of pyrethroids are already available on the market.

The main current alternative to IRS is the use of ITNs. The insecticide enhances the protective effect for the person under the net, but also has a beneficial effect on the community at large (Hawley et al. 2003). ITNs have been shown convincingly to cause substantial reductions in all-cause child mortality, under both experimental (Lengeler 2004) and operational conditions (Armstrong Schellenberg et al. 2001; Fegan et al. 2007). They are effective in highly endemic settings by reducing the risk of severe disease, particularly in infants and young children before they have acquired a certain level of natural immunity (Smith et al. 2001). Two categories of ITNs are available: conventionally treated nets and long-lasting ITNs. The former needs regular retreatment, a follow-up action that has proven difficult to achieve at field level. The latter is a relatively new technology that retains the efficacy for at least 3 years. Pyrethroids are the only chemical group recommended for use in ITNs.

There have been several new developments in ITN technology. Research on treatment with nonpyrethroids has been conducted to cope with the problem of resistance, but safety issues are a concern. At least one insecticide with novel chemistry is being developed for ITNs (Hemingway et al. 2006). It is critical that this unique product, once it enters the market, is reserved solely for public health purposes, thus reducing the risk of insecticide resistance in the future. New ITN products are not expected to come to market in the short term.

The relative cost-effectiveness of IRS and ITNs has been studied on several occasions. Both have been considered attractive interventions in terms of cost per disability-adjusted life-years averted (Goodman et al. 2000), but their relative effectiveness depends on vector behavior and human sleeping habits in a given setting. ITNs are generally more cost-effective in highly endemic settings (Yukich et al. 2008), whereas IRS operations can respond faster to epidemic situations (Curtis and Mnzava 2000).

The use of chemical insecticides as larvicides to control mosquito breeding can play an important role in malaria control where this is appropriate and feasible, particularly in urban settings, but the broad-spectrum effects of most chemicals are a concern to the integrity of aquatic ecosystems. Moreover, chemical repellents could have a useful supplementary role in vector control (Rowland et al. 2004). Innovative work is in progress on the attractiveness of human odors to malaria vectors, with potential applications as mosquito attractants and repellents for use in trapping and personal protection (Zwiebel and Takken 2004).

### Nonchemical methods

“Environmental management for vector control” is the collective term for manipulating or modifying environmental factors or their interaction with humans to reduce vector breeding and vector–human contact. Before the advent of synthetic insecticides, vector control depended primarily on environmental management; a meta-analysis of data mostly from that period indicated that it substantially reduced malaria risk (Keiser et al. 2005). Eliminating vector-breeding habitats and managing water bodies has the potential to suppress vector populations, particularly in human-made habitats or urban settings (Walker and Lynch 2007). In irrigated agriculture, vector breeding can be controlled, for example, through land leveling and intermittent irrigation (Keiser et al. 2002). New irrigation systems or dams cause drastic changes in vector–human contact, and planning to avoid health risks is essential at the design stage.

Improvement of housing, for example, through plastering of walls or closing of eaves, contributes significantly to transmission control (Gunawardena et al. 1998). Moreover, screening to keep mosquitoes out at night is a protective option for houses with solid walls (Lindsay et al. 2002). However, information on the cost and feasibility of housing improvement in various settings is largely missing.

The role of aquatic predators as control agents of malaria vectors is potentially enhanced through conservation or through the introduction of agents from outside. Larvivorous fish have frequently been reared and released for controlling vector breeding in small water tanks and wells, but successes have generally been limited to more or less permanent water bodies (Walker and Lynch 2007).

The bacteria *Bacillus thuringiensis israelensis* and *Bacillus sphaericus* are used in formulations as microbial larvicides. They produce toxins that are specific to mosquitoes and that have a low risk of resistance development (Lacey 2007). Recent field trials and pilot projects have shown good potential of both bacteria to manage mosquito breeding and to reduce biting rates in certain settings (Fillinger et al. 2008). Insect pathogenic fungi have shown promising results for controlling adult *Anopheles* mosquitoes when sprayed on indoor surfaces and have potential to substantially reduce malaria transmission (Scholte et al. 2005). Other alternative vector control methods include the use of locally available plants or plant materials as mosquito repellents or as larvicides (Okumu et al. 2007; Seyoum et al. 2003), and the use of expanded polystyrene beads in specific breeding sites (Yapabandara and Curtis 2002). Novel methods under development are genetically engineered mosquitoes and the sterile insect technique (Catteruccia 2007).

Data on the cost-effectiveness of non-chemical methods are scarce. In a retrospective analysis of data from Zambia, Utzinger et al. (2001) indicated that environmental management was as cost-effective as ITNs. Moreover, environmental management can benefit from local resources, reducing the need for external funds.

### Current implementation of DDT alternatives

The past decade has seen a steady increase in commitment to malaria control by the international community (Snow et al. 2008). This has caused a boost in financial and human resources available for implementation of vector control interventions, due to the support of the Global Fund, the World Bank, the U.S. President’s Malaria Initiative, and many nongovernmental organizations.

China, the Solomon Islands, and Vietnam have largely replaced their IRS programs with ITNs during the past decades (Najera and Zaim 2001). Conversely, the use of IRS is on the increase in Africa, where it has been more difficult to come to grips with malaria because of aspects of vector biology and disease epidemiology. In South Asia, indoor spraying using DDT and alternative insecticides continues on a large scale, but the quality of the intervention is a critical issue (JMM 2007).

National campaigns of free or highly subsidized ITNs, often in combination with other malaria control interventions, have reportedly approached coverage levels of ≥ 50% among households in a number of African countries, resulting in dramatic reductions in the malaria incidence (Bhattarai et al. 2007; Nyarango et al. 2006; Otten et al. 2009; WHO 2008b).

Nonchemical methods, such as environmental management and biological control have been promoted or tested in pilot projects. However, contemporary cases of sustained implementation are not common. Case examples include the use of intermittent irrigation in China (Liu et al. 2004), integrated and participatory strategies in Mexico (Chanon et al. 2003) and India (Sharma 1987), river flow management in Sri Lanka (Konradsen et al. 1998), and the use of farmer field schools on vector management in agriculture in Sri Lanka (van den Berg et al. 2007).

### Barriers and gaps

Several barriers exist in the implementation of alternatives to DDT. Vector resistance to insecticides is a direct threat to the sustainability of ITNs and IRS. Resistance to pyrethroids has been reported in malaria vectors from West, East, and southern Africa (ANVR 2005; Coleman et al. 2007). Particularly, *kdr*-type cross-resistance between pyrethroids and DDT severely limits the choice of insecticide. South Africa was forced to reintroduce DDT after failure of pyrethroids, due to one of the locally extinct vectors returning and having acquired pyrethroid resistance (not *kdr*-type) elsewhere (Hargreaves et al. 2000).

There is growing concern about sustained effectiveness of ITNs because the intervention currently depends solely on pyrethroid insecticides (Greenwood et al. 2008). Multivillage studies in an area with highly resistant *A. gambiae* in Côte d’Ivoire indicated that ITNs retained most of their effect (Chandre et al. 2000; Henry et al. 2005). The explanation for this finding was that resistant mosquitoes were less irritated, which resulted in a higher uptake of insecticide. More worrisome are the results of a semi–field study from an area with highly resistant vectors in Benin (N’Guessan et al. 2007b), which showed a major loss in efficacy of ITNs locally. Without the insecticidal action, bed nets provide a much lower level of personal protection (Lengeler 2004).

Resistance is caused by the use of insecticides in agriculture (Diabate at al. 2002) and in public health. There is evidence of increased frequencies of resistance genes attributable to IRS or ITN programs (Karunaratne and Hemingway 2001; Stump et al. 2004). Moreover, there are records of a change in vector behavior from indoor resting to outdoor resting in response to indoor spraying, as well as a change in daily pattern of biting and host choice in response to ITN interventions (Molineaux and Gramiccia 1980; Pates and Curtis 2005; Phillips 2001; Takken 2002). A system of sentinel sites to monitor vector density, quantify insecticide resistance, and guide informed decision making on insecticide choice still needs to be established in most disease-endemic countries (Coleman and Hemingway 2007).

Another barrier is operational capacity. The effective coverage of programs depends critically on the access and targeting of populations and vulnerable groups most at risk of malaria, the degree of compliance of the provider, and adherence by the consumer. In most countries with endemic malaria, health systems lack capacity to plan and implement programs effectively. Reforms in the health sector have led to the decentralization of planning and budgeting. Consequently, the responsibility for service provision has shifted from national to subnational or district-level health departments, requiring new skills for malaria control at each level. An analysis of case studies from four countries suggested that decentralization can potentially benefit malaria control (Barat 2006). In general, however, there is a lack of guidance on how malaria control might be implemented in a decentralized environment (World Bank 2005).

Traditionally, IRS has been managed as vertical programs, which is still the case in various countries. In some countries the transition process after health reforms has caused an erosion of the specialist skills needed for IRS (Shiff 2002). It will be a challenge for many countries to conduct and sustain effective IRS programs (Kolaczinski et al. 2007). The delivery of ITNs has used a variety of models, including vertical programs, integrated health sector programs, and involvement of the private sector and nongovernmental organizations (Webster et al. 2007). As the global thrust is to promote coverage with ITNs and IRS, vector control capacity is needed at the appropriate levels.

Interventions involving environmental management and other larval control methods depend on the participation of other sectors and communities. Even though decisions affecting the risk of vector-borne disease are taken in other public sectors, there is insufficient awareness of the effects. Moreover, the health sector lacks capacity to facilitate community participation and education. A possible solution is the integration of health activities with community programs that generate income (e.g., from agriculture). Rich experience with participatory approaches exists within the agriculture sector (Pretty 1995); the health sector potentially can benefit from these resources. One relevant model is the Farmer Field School on Integrated Pest Management, developed and promoted by the Food and Agriculture Organization of the United Nations (van den Berg and Knols 2006).

### Integration of methods

An integrated approach to vector control has frequently been advocated (McKenzie et al. 2002; Shiff 2002; Utzinger et al. 2002). The need for a reduced reliance on insecticides for vector- borne disease control, as pointed out in World Health Assembly Resolution 50.13 (International Programme on Chemical Safety 1997), has been stressed further by the Intergovernmental Forum on Chemical Safety, Forum VI (Intergovernmental Forum on Chemical Safety 2008).

Various studies have demonstrated that integration of vector control methods resulted in significant reductions in transmission and morbidity rates of malaria (Chanon et al. 2003; Dua et al. 1997; Sharma et al. 1991; Singh et al. 2006; Takken et al. 1990; Utzinger et al. 2001). Moreover, modeling studies predicted that combinations of interventions can be much more effective in reducing malaria transmission than individual interventions and that the effect of IRS and ITNs is amplified by environmental management, even in areas of intense transmission (Killeen et al. 2000, 2004).

Besides its direct effect on transmission intensity, the integration of methods may also contribute to resistance management. For example, larval control is expected to prevent or delay the onset of vector resistance to insecticides (Walker and Lynch 2007), whereas measures that reduce human contact with vectors, through their proximity, housing conditions, or presence of repellents, for example, will reduce the selection pressure.

### Integrated vector management

Modeled on the positive experience from integrated pest management in agriculture, integrated vector management (IVM) has been defined by the WHO (2008a) as “a rational decision-making process for the optimal use of resources for vector control.” The aim of IVM is to improve cost-effectiveness, ecologic soundness, and sustainability of disease vector control (Townson et al. 2005; WHO 2004). In contrast to conventional vector control programs with a top-down decision-making structure, IVM emphasizes decision making at the lowest possible level in accordance with local data collection and situational analysis, and requires collaboration within the health sector and with other sectors, as well as community participation. Hence, decentralization in the health sector can potentially work in favor of IVM by facilitating tailored action at the local level (van den Berg and Takken 2007).

The Global Malaria Action Plan advocates the scaling-up of ITNs and IRS for an immediate impact on the malaria burden of populations at risk (Roll Back Malaria Partnership 2008). However, to address sustainability issues, interventions must be implemented in accordance with an IVM approach by being evidence-based and by integrating available resources and supplementary methods in an effective and ecologically sound manner. To enable the graduation from a conventional vector control program to IVM, the evidence base and human capacity needs strengthening at all relevant levels of administration. Recently, targets have been set for the elimination of malaria (Feachem and Sabot 2008). An IVM approach is important to sustain achievements and reduce transmission to critical low levels needed to eliminate malaria (Beier et al. 2009).

## Conclusions

The reported global use of DDT for disease vector control is 4–5,000 metric tons per year, with India by far the largest consumer and several countries reintroducing DDT. The insecticide is known for its long residual effect and low operational cost. However, the effectiveness of DDT depends on local settings and merits closer consideration vis-à-vis chemical and nonchemical alternatives. Legislation and capacity to enforce regulations and management practice is inadequate in most countries.

Recent evidence indicates that indoor spraying causes high levels of human exposure to DDT (e.g. Aneck-Hahn et al. 2007). This could adversely affect human health, because the evidence base on some of the more serious and chronic health effects of DDT is growing. Moreover, the occurrence of resistance to the toxic action of DDT is common in malaria vectors and appears to be spreading. A comprehensive cost assessment of DDT versus its alternatives is needed and should include the monitoring of side effects and unintended costs to human health, the environment, and international trade.

Effective chemical alternatives to DDT for vector control are available, but the choice of insecticides is limited. Insecticides with novel chemistry will not come to market in the short term. Alternative insecticides should pose less risk to human health and the environment. The coverage of populations with ITNs and IRS has increased in recent years, particularly in Africa. However, insecticide resistance is reducing the efficacy of these methods in certain areas. To be prepared for future emergencies, the continued effectiveness of insecticides needs to be safeguarded.

A number of nonchemical methods have proven their value in malaria control in certain settings, but more work is needed on the incremental impact of methods such as environmental management or the use of microbial larvicides when used in conjunction with IRS and ITNs. Several new technologies are under development but require increased investment. To continue this development, we must foster new researchers in the field of vector control.

To reduce reliance on DDT, support is needed for integrated and multipartner strategies of vector control. IVM provides a framework for improving cost-effectiveness, ecologic soundness, and sustainability of vector control through integration with other arms of public health and other sectors. Now that malaria transmission is decreasing in a number of African countries, there is a greater prospective role for environmental management and other nonchemical methods within IVM strategies. This will increase the sustainability of control efforts and assist in achieving malaria elimination objectives.

## Figures and Tables

**Table 1:** Annual global production and use of DDT (in 10^3^ kg active ingredient) in 2003, 2005, and 2007.

Country	2003	2005	2007	Comment	Source
Produce DDT for vector control					
China^a^	450	490	NA	For export	Pd
India^b^	4,100	4,250	4,495	For malaria and leishmaniasis	Pd, Ws, Dc
DPRK	NA	NA	5	> 155 metric tons for use in agriculture	UNITAR
Global production	> 4,550	> 4,740	> 4,500		

Use DDT for vector control					
Cameroon	0	0	0	Plan to pilot in 2009	WHO
China	0	0	0	Discontinued use in 2003	SC
Eritrea	13	15	15	Epidemic-prone areas	Qu, WHO
Ethiopia	272	398	371	Epidemic-prone areas	WHO, Ws
Gambia	0	0	NA	Reintroduction in 2008	Dc
India	4,444	4,253	3,413	For malaria and leishmaniasis	WHO, Dc
DPRK	NA	NA	5	> 155 metric tons used in agriculture	UNITAR
Madagascar	45	0	0	Plan to resume use in 2009	Qu
Malawi	0	0	0	Plan to pilot in 2009	WHO
Mauritius	1	1	< 1	To prevent malaria introduction	Qu
Morocco	1	1	0	For occasional outbreaks	Qu
Mozambique	0	308	NA	Reintroduction in 2005	WHO
Myanmar	1	1	NA	Phasing out	Ws
Namibia	40	40	40	Long-term use	WHO
Papua New Guinea	NA	NA	0	No recent use reported	SC
South Africa	54	62	66	Reintroduction in 2000	Qu, WHO
Sudan	75	NA	0	No recent use reported	Qu, WHO
Swaziland	NA	8	8	Long-term use	WHO
Uganda	0	0	NA	High Court prohibited use, 2008	SC, Dc
Zambia	7	26	22	Reintroduction in 2000	Ws, Qu, WHO
Zimbabwe	0	108	12	Reintroduction in 2004	WHO
Global use	> 4,953	> 5,219	> 3,950		

Abbreviations: Dc: Direct communication with national authorities; NA, not available; Pd: project proposals submitted to the Global Environment Facility; Qu: questionnaire on DDT by the Secretariat of the Stockholm Convention completed by national autorities; SC: documents published by the Secretariat; Ws: workshop presentations by country delegates in the context of the Stockholm Convention. Further information was obtained from the WHO and UNITAR reports, as indicated.

a The figure for 2005 was extrapolated from the total production; in addition to production for vector control, DDT is produced for Dicofol manufacture (~ 3,800 metric tons per year) and for antifoulant paints (~ 200 metric tons per year).

b DDT is also produced for dicofol manufacture (~ 280 metric tons per year).

**Table 2:** Alternative methods for malaria vector control, indicating the targeted vector stage, the potential risk, and required resources and delivery mechanisms.

Vector management method	Vector stage	Risk^a^	Resources/delivery
Chemical methods			
Insecticide-treated bed nets	Adult	Resistance, toxicity	Free distribution, social marketing, private sector
Indoor residual spraying	Adult	Resistance, toxicity	Spray teams
Chemical larviciding	Larva	Resistance, effect on ecosystems	Spray teams
Repellents and attractants^b^	Adult	Toxicity	Local, private sector

Nonchemicals methods			
Elimination of breeding sites	Larva	—	Local
Habitat manipulation	Larva	—	Local, agriculture sector
Irrigation management	Larva	—	Local, irrigation sector
Design of irrigation structures	Larva	—	Irrigation sector
House improvement	Adult	—	Local, development programs
Predation	Larva	—	Local, programs, agriculture sector
Microbial larvicides	Larva	Resistance	Programs, private sector
Botanicals	Larva/adult	Toxicity	Local
Polystyrene beads	Larva	—	Local
Fungi^b^	Adult	—	Not applicable
Genetic methods^b^	Adult	To be studied	Not applicable

—, Negligible risk.

a Theoretically, (behavioral) resistance could also develop against repellents, attractants, and house improvement.

b (Partly) under development.
